# Transcriptomic profile of premature ovarian insufficiency with RNA-sequencing

**DOI:** 10.3389/fcell.2024.1370772

**Published:** 2024-04-09

**Authors:** Jiaman Wu, Shiyu Feng, Yan Luo, Yan Ning, Pingping Qiu, Yanting Lin, Fei Ma, Yuanyuan Zhuo

**Affiliations:** ^1^ Department of Chinese Medicine, Shenzhen Maternity and Child Healthcare Hospital, Shenzhen, China; ^2^ Guangzhou University of Chinese Medicine, Guangzhou, China; ^3^ Department of Acupuncture and Moxibustion, Shenzhen Traditional Chinese Medicine Hospital, Shenzhen, China

**Keywords:** premature ovarian insufficiency, transcriptomic, genes, biomarker classification, RNA-sequencing

## Abstract

**Introduction:**

This study aimed to explore the transcriptomic profile of premature ovarian insufficiency (POI) by investigating alterations in gene expression.

**Methods:**

A total of sixty-one women, comprising 31 individuals with POI in the POI group and 30 healthy women in the control group (HC group), aged between 24 and 40 years, were recruited for this study. The transcriptomic profiles of peripheral blood samples from all study subjects were analyzed using RNA-sequencing.

**Results:**

The results revealed 39 differentially expressed genes in individuals with POI compared to healthy controls, with 10 upregulated and 29 downregulated genes. Correlation analysis highlighted the relationship between the expression of SLC25A39, CNIH3, and PDZK1IP1 and hormone levels. Additionally, an effective classification model was developed using SLC25A39, CNIH3, PDZK1IP1, SHISA4, and LOC389834. Functional enrichment analysis demonstrated the involvement of these differentially expressed genes in the “haptoglobin-hemoglobin complex,” while KEGG pathway analysis indicated their participation in the “Proteoglycans in cancer” pathway.

**Conclusion:**

The identified genes could play a crucial role in characterizing the genetic foundation of POI, potentially serving as valuable biomarkers for enhancing disease classification accuracy.

## Introduction

Premature ovarian insufficiency (POI) is a condition characterized by the depletion of ovarian function before the age of 40 (Nelson, 2009a), presenting a significant reproductive challenge and serving as a prevalent cause of female infertility (Welt, 2008). Recent data reveal a concerning rise in POI incidence, impacting approximately six million Chinese women before the age of 40 (Lin et al., 2017). The etiologies of POI are notably diverse, with over half of the cases presenting as complex and uncertain. Identified causative factors encompass spontaneous genetic defects, autoimmune diseases, infections, or iatrogenic interventions (De Vos et al., 2010). Genetic factors, a well-established contributor, account for about 20%–25% of cases, underscoring the need for robust research to unravel pathogenic mechanisms and identify therapeutic targets (Qin et al., 2015; França and Mendonca, 2022). This pursuit is crucial for facilitating early diagnosis and the development of effective treatments for POI.

Recent advancements in high-throughput sequencing technology have emerged as crucial tools for delving into the origins of POI. While the exact mechanisms underlying POI remain incompletely understood, significant strides have been made in identifying genes that play a role in its development (Jaillard et al., 2020). Currently, approximately 90 genes have been associated with either isolated or syndromic forms of POI, underscoring the substantial genetic diversity inherent in the condition (Dixit et al., 2010). Gene expression analyses have shed light on the molecular underpinnings driving the pathogenesis of POI. In a comprehensive study, Smith et al. delved into gene expressions in ovarian tissue from POI patients, revealing disruptions in crucial signaling pathways that govern follicular development and hormonal regulation (Simoni and Casarini, 2014). Noteworthy genes such as FSHR and BMP15 were implicated, highlighting their vital roles in maintaining normal ovarian function (Simoni and Casarini, 2014). Additionally, Johnson et al.'s recent research explored the epigenetic regulation of POI-associated genes, revealing irregular DNA methylation patterns (Di-Battista et al., 2023). This sheds light on potential epigenetic mechanisms contributing to POI’s progression. Furthermore, a mouse study utilizing transcriptome sequencing revealed that the decreased mRNA and protein expression levels may contribute to an increase in N6-methyladenosine, potentially elevating the risk of POI-related complications (Ding et al., 2018). These epigenetic insights offer a fresh outlook on how gene expression disturbances might underlie POI’s origins. As ongoing research continues to unveil the intricate genetic aspects of POI, there is promising potential for advancing diagnostics, treatment, and management strategies for individuals affected by this condition. Recognizing the significant variability in gene expression patterns among different ethnic groups (Ishizuka, 2021), it is crucial to delve into specific populations for a more comprehensive understanding. With this consideration, this article narrows its focus to the Chinese population, acknowledging the importance of recognizing potential ethnic-specific factors that could influence the development of POI.

The aim of this study was to explore the physiological significance of ovarian dysfunction in individuals diagnosed with POI using peripheral blood transcriptome sequencing. Spontaneous POI cases were meticulously selected, and healthy female subjects were matched 1:1 from clinical settings. Transcriptome gene sequencing was employed to characterize the functional attributes and potential roles of differentially expressed genes in peripheral blood samples. This research endeavor provides a valuable opportunity to systematically explore the genetic landscape of POI, enhancing our ability to identify relevant genetic factors. Understanding the intricate interplay between genes and ovarian function in POI is crucial for effectively managing and potentially improving ovarian failure.

## Materials and methods

### Participants, study design and sample collection

A total of sixty-one women, aged between 24 and 40 years, were recruited from Shenzhen Maternity and Child Healthcare Hospital between January and March 2023. Among them, thirty-one women were diagnosed with spontaneous Premature Ovarian Insufficiency (POI), while thirty served as healthy controls. The diagnostic criteria for POI were consistent with prior reports (Guo et al., 2020), including primary or secondary amenorrhea for at least four consecutive months before the age of 40, coupled with at least two instances of serum follicle-stimulating hormone (FSH) levels exceeding 40 IU/L, measured 4–6 weeks apart. Control participants were selected to closely match the POI group in terms of age, lifestyle, and medical history. They had regular menstrual cycles and FSH levels below 10 IU/L, indicating normal ovarian function, with no history of menstrual dysfunction or infertility. Individuals with the following conditions were excluded: chromosomal abnormalities, history of ovarian surgery, family history of POI, pregnancy or lactation, serious diseases requiring prolonged medical treatment, chronic diarrhea, autoimmune diseases, recent use of hormone replacement treatment within the last 3 months, gastrointestinal disease, active infections, body mass index (BMI) below 18.5 or above 23.9 kg/m2, smoking, or undergoing chemotherapy or radiotherapy. Relevant clinical characteristics were extracted from participants’ health records.

The study protocol was approved by the ethics committee of Shenzhen Maternity and Child Healthcare Hospital, with the approval number SFLYS[2022]058. Prior to enrollment, written informed consent was obtained from all participants, ensuring their voluntary participation in the study.

Participants underwent venous blood collection on the 2nd-3rd day of a menstrual cycle. In cases of amenorrhea, blood samples were randomly obtained for examination. Peripheral blood samples were collected from all participants following standardized protocols to minimize variability in sample process and collected in the morning after an overnight fast to minimize the influence of diurnal variations in gene expression. These samples were utilized for the examination of sex hormones and extraction of RNA, which served as the basis for subsequent analyses. Electron chemiluminescence immunoassay was used to detect the levels of FSH, E2, and AMH.

### RNA extraction, library preparation and sequencing

RNA extraction was conducted using TRIzol reagent (Invitrogen, Carlsbad, CA, USA), following the manufacturer’s instructions. The quality and concentration of the extracted RNA were assessed using a Nanodrop instrument, while the RNA integrity was evaluated using the Agilent 2,100 Bioanalyzer (Agilent, CA, USA). Only samples with RNA integrity number (RIN) greater than seven were deemed suitable for further RNA library constitution and subsequent analyses.

Library construction was carried out using Illumina TruSeq Stranded mRNA Library Prep Kit (Illumina, CA, United States), following the manufacturer’s instructions. The process involved several steps. Firstly, mRNA molecules possessing polyA tails were enriched using magnetic beads, thereby eliminating rRNA and other non-coding RNA species. Subsequently, reverse transcription was conducted using random hexamer primers, resulting in the synthesis of the first strand of cDNA. DNA polymerase was then employed to synthesize the second strand of cDNA, thereby generating double-stranded cDNA molecules. The double-stranded cDNA underwent end repair and A-tailing, followed by ligation of Illumina TruSeq index adapters. Finally, PCR amplification was carried out to enrich the library, and the quality and size distribution of the library were assessed using the Agilent 2,100 Bioanalyzer.

Sequencing was conducted on the Illumina NovoSeq 6,000 platform, employing paired-end 150 bp reads. The resulting sequencing data were saved in FASTQ format. To ensure data quality, FastQC software was utilized to perform quality control analysis.

### Bioinformatic and statistical analysis

For quantifying the RNA-seq data, we employed the kallisto software, utilizing its pseudo-alignment algorithm for rapid estimation of transcript relative abundance (Bray et al., 2016). Differential expression analysis was conducted using the DESeq2 package (Love et al., 2014) which incorporates a negative binomial distribution model and data-driven prior distributions to effectively control for technical and biological variation. This approach provides robust *p*-values and multiple testing corrected *p*-values, ensuring reliable statistical analysis. Enrichment analysis of differentially expressed genes was performed using the cluster Profiler package (Yu et al., 2012).

The Shapiro-Wilk test was employed to examine the distribution types of continuous variables. Continuous variables that followed a normal distribution were presented as mean ± standard deviation (SD) and compared between groups using Student’s *t*-test.

Pearson analysis was employed to examine the relationships between gens expression and clinical indicators associated with ovarian reserve, ovarian endocrine function, and perimenopausal syndrome symptoms. Furthermore, for genes exhibiting significant differences, receiver operating characteristic (ROC) curves were constructed, and the corresponding area under the curve (AUC) values were computed. Statistical significance was set at a two-tailed *p*-value of less than 0.05. All data analyses were performed using R software.

## Results

### Participant characteristics

A total of 31 women with POI and 30 healthy controls were included in the analysis. Table 1 provided an overview of the clinical characteristics of the two groups. The mean ages in the POI group was 36.22 ± 3.13, while in the control, it was 35.04 ± 3.33 years. There were no significant differences in age between the two groups. Additionally, the mean body mass index (BMI) in the POI was 21.57 ± 0.08, while in the control group, it was 21.75 ± 1.30. BMI did not significantly differ between the two groups. In terms of hormonal profiles, women with POI exhibited significantly higher levels of follicle-stimulating hormone (FSH), luteinizing hormone (LH), and testosterone compared to the control group. Moreover, the FSH/LH ratio was also significantly higher in the POI group. Conversely, anti-Müllerian hormone (AMH) levels were significantly lower in women with POI compared to the control group. There was no statistically significant difference in the history of childbirth and the number of pregnancies between the two groups, but there was a statistically significant difference in the number of AFC detected by ultrasound (*p* < 0.05).

**TABLE 1:** Demographic and clinical characteristics of the two groups.

	POI group (n = 31)^a^	Control group (n = 30)^a^	*p-*value
Age (years)	36.22 ± 3.13	35.04 ± 3.33	0.17
BMI (kg/m^2^)	21.57 ± 0.88	21.75 ± 1.30	0.52
FSH (mIU/mL)	41.44 ± 21.36	5.23 ± 1.21	<0.01**
LH (mIU/mL)	17.07 ± 8.10	4.42 ± 0.79	<0.01**
E2 (pg/L)	34.27 ± 11.10	45.37 ± 4.37	<0.01**
AMH (ng/mL)	0.41 ± 0.30	2.60 ± 0.53	<0.01**
FSH/LH (ratio)	2.41 ± 0.60	1.19 ± 0.21	<0.01**
No history of childbirth (%)	20 (64.51)	13 (43.33)	0.09
Number of pregnancies			0.72
0	18 (58.06)	15 (50.00)	
1	4 (12.90)	6 (20.00)	
≥2	9 (29.03)	9 (30.00)	
AFC	5.35 ± 2.10	12.95 ± 2.30	<0.01**

POI, premature ovarian insufficiency, BMI, body mass index, FSH, Follicle-stimulating hormone, LH, luteinizing hormone, E2: oestradiol, AMH, Anti-Müllerian hormone. AFC, antral follicle count,*When *p* < 0.05, “*” marks the significant, **When *p* < 0.01, “**” marks significance.

^a^ Normally distributed data are expressed as means ± standard deviations.

### Identification of differentially expressed genes

**FIGURE 1:**
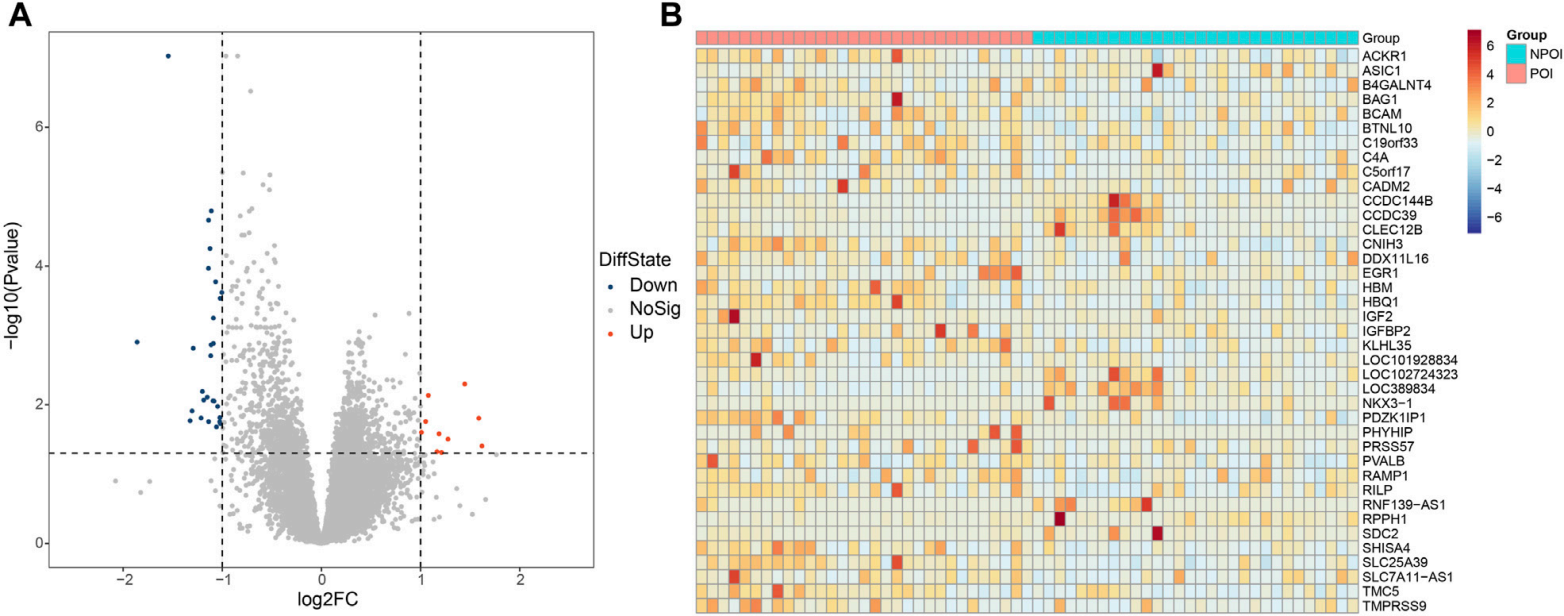
Differentially expressed genes between the POI and control groups. **(A)** Volcano plot illustrating the differentially expressed genes. A total of 39 genes were found to be differentially expressed, with 29 genes showing downregulation (FC < −2 and p.adjust <0.05) and 10 genes exhibiting upregulation (FC > 2 and p.adjust <0.05). Upregulated genes are represented by red dots, downregulated genes are denoted by blue dots, and genes with no significant changes are indicated by the grey dots. **(B)** Heatmap depicting the expression patterns of the differentially expressed genes. The color key from red to blue, representing low to high relative gene expression levels, respectively.

Differential gene expression analysis between the POI and control groups was performed using the DESeq2 software. Genes were considered differentially expressed if they exhibited a fold change greater than two and an adjust *p*-value (p.adjust) less than 0.05. As a result, we identified 39 genes that were differentially expressed in women with POI compared to control group. Among these genes, 10 were upregulated, while 29 were downregulated (Figure 1A). The expression levels of these genes in each sample were depicted in Figure 1B.

To explore the pathophysiological relevance of the differentially expressed genes, we conducted functional enrichment analysis. GO analysis, specifically focusing on the molecular category, revealed significant enrichment of the differentially expressed genes in haptoglobin binding and oxygen carrier activity (Table 2). Additionally, in the GO category of cellular components, these genes were found to be enriched in the haptoglobin-hemoglobin complex and hemoglobin complex (Table 2). Furthermore, KEGG pathway enrichment analysis indicated the involvement of the differentially expressed genes in the “Proteoglycans in cancer” pathway (Table 2).

**TABLE 2:** Significant enrichment of GO terms and pathways.

Group	ID	Description	*p-*value
cellular components	GO:0031838	haptoglobin-hemoglobin complex	<0.01**
	GO:0005833	hemoglobin complex	<0.01**
molecular function	GO:0031720	haptoglobin binding	<0.01**
	GO:0005344	oxygen carrier activity	<0.01**
KEGG	hsa05205	Proteoglycans in cancer	<0.05*

**FIGURE 2:**
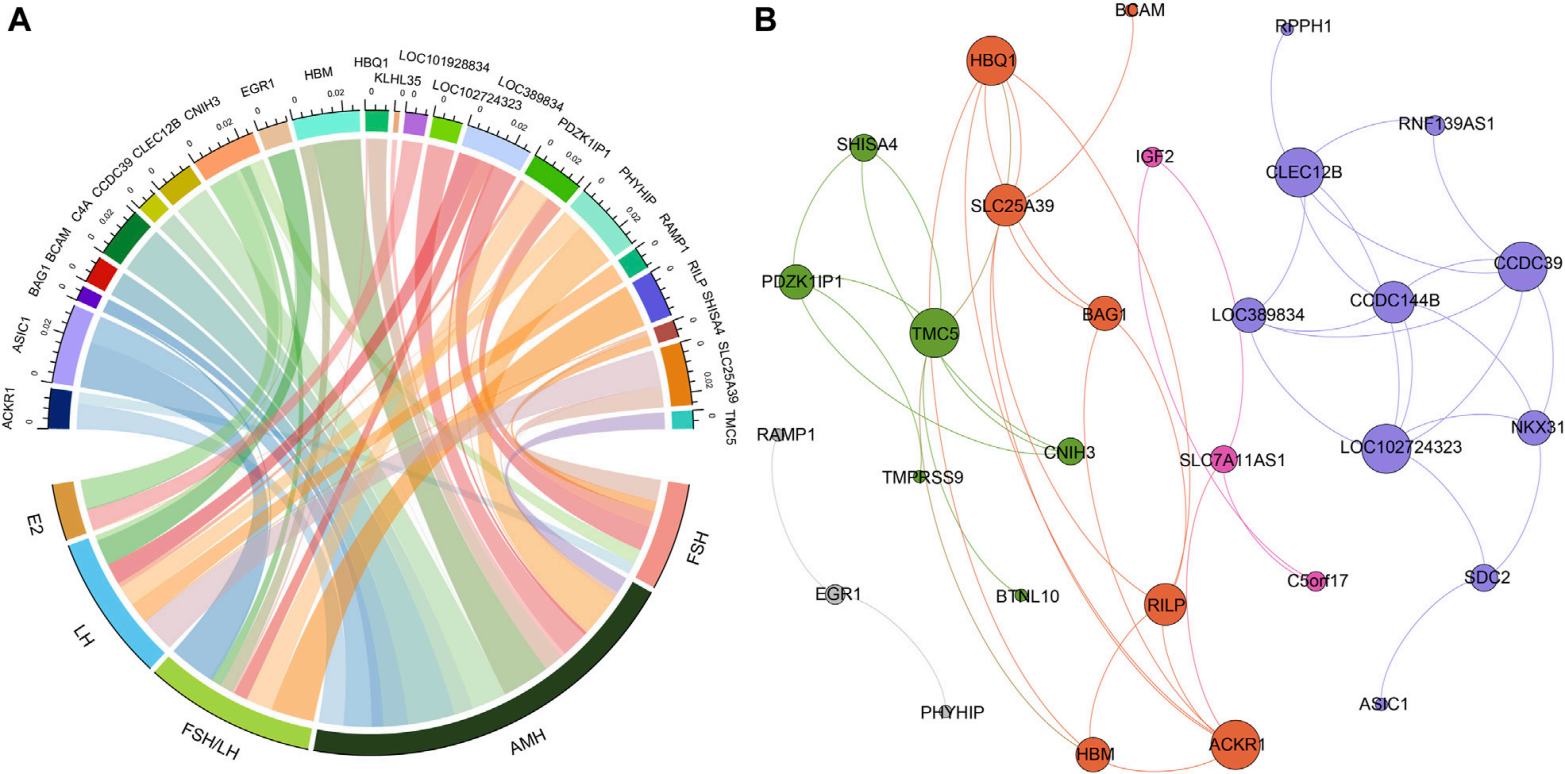
The relationships between hormone levels and differentially expressed genes. **(A)** The correlations between hormone levels and differentially expressed genes. **(B)** The correlations among the differentially expressed genes.

To assess the potential effectiveness of classifying POI based on the differentially expressed genes, we utilized support vector machine algorithm with 5-fold cross-validation to construct a classification mode. The receiver operating characteristic curve of the mode was depicted in Figure 2, demonstrating the AUC value of 0.83. Moreover, when we selected top five differentially expressed genes, SLC25A39, CNIH3, PDZK1IP1, SHISA4, and LOC389834, the AUC improved to 0.88. When considering hormone levels in addition to the top five gene expression profiles, the AUC reached 1. Remarkably, even using hormone levels alone as predictors, the AUC remained 1.

### Correlation of differentially expressed genes and hormone levels

**FIGURE 3:**
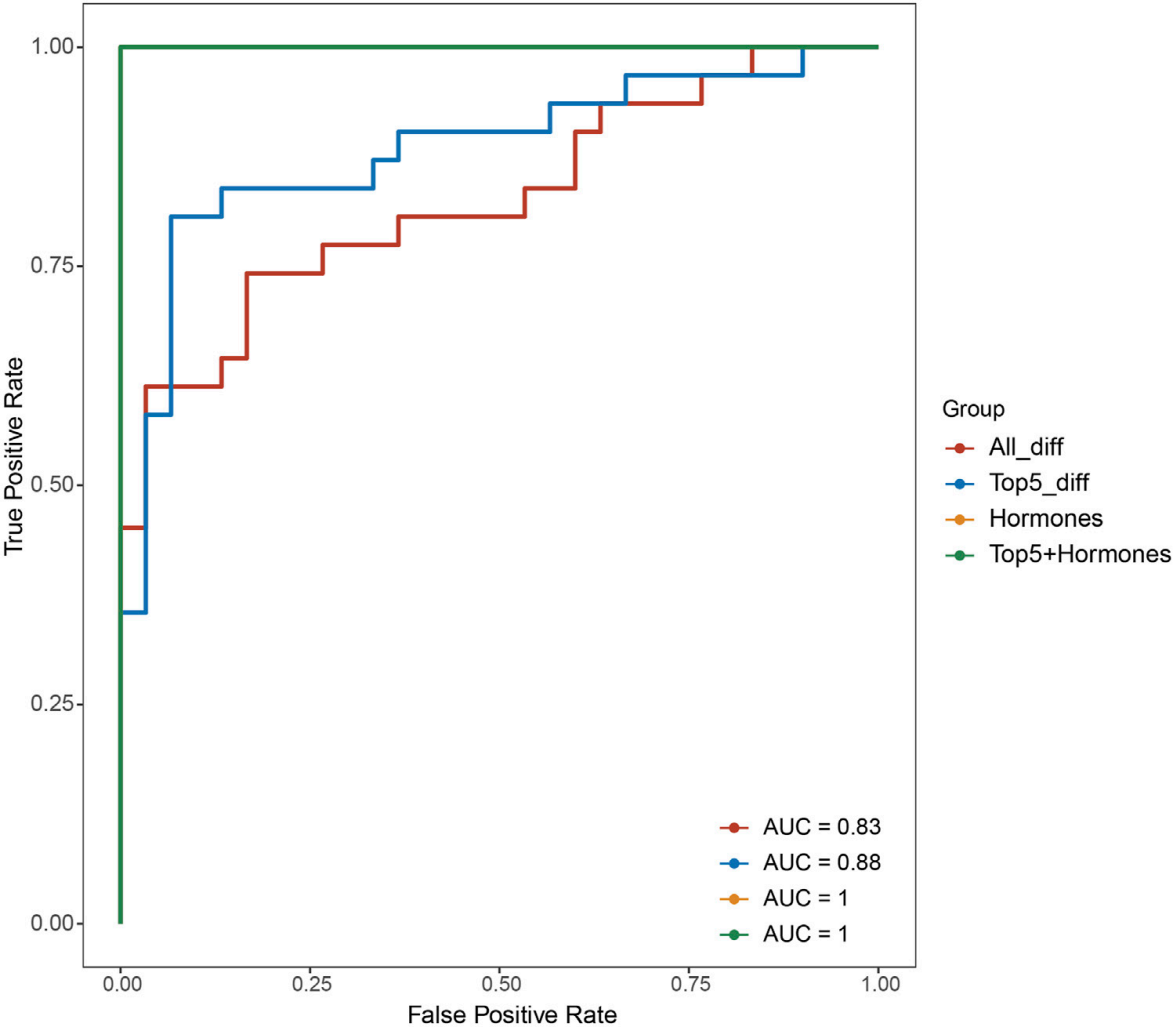
Receiver operating characteristic curves of different classification model based on different feature sets (all differentially expressed genes, top five differentially expressed genes, hormone levels, top five differentially expressed genes and hormones combined).

Figure 3A presented the results of Pearson correlation analysis, which was performed to investigate the relationships between the differentially expressed genes and hormone levels. To determine significant correlations, a threshold of *p*-value <0.05 and absolute *R* ^2^ value exceeding 0.30 were applied. The correlation analysis revealed noteworthy associations between the differentially expressed genes and hormone levels. Specifically, AMH level exhibited positive correlations with five genes and negative correlations with thirteen genes. Conversely, E2 level displayed negative correlations with four genes. FSH level exhibited positive correlation with five genes and negative correlations with one gene. LH level demonstrated positive correlations with seven genes and negative correlations with one gene. Moreover, the FSH/LH ratio displayed positive correlations with eight genes and negative correlations with two genes. Of particular interest, the gene CNIH3 exhibited correlations with all hormone levels investigated. Additionally, genes PDZK1IP1 and SLC25A39 showed associations with FSH, LH, AMH, and FSH/LH levels.

### Correlations between differentially expressed genes

In addition, we investigated the correlations between the differentially expressed genes, as illustrated in Figure 3B. Through modularity class analysis, we identified five distinct clusters. Cluster one consisted of 10 genes, represented by purple nodes. Cluster two comprised seven genes, represented by orange nodes. Cluster three included six genes, represented by green nodes. Cluster four encompassed three genes, represented by pink nodes. Lastly, cluster five contained three genes. Within cluster 1, CCDC39, CLEC12B, and LOC102724323 exhibited the highest number of edges, indicating a strong interconnectedness among these genes. In cluster 2, the prominent genes were ACKR1 and HBQ1. TMC5 was found to be the dominant gene in cluster 3.

## Discussion

Premature ovarian insufficiency occurs when there is a premature depletion of oocytes, resulting in a decline in ovarian activity before the age of 40 (Takahashi et al., 2021). This decline is often preceded by a decrease in the number of residual follicles and a reduction in ovarian reserve capacity (Goldman et al., 2017). Therefore, a decrease in ovarian reserve capacity serves as a precursor to the development of POI. The causes of POI are diverse and can include genetic abnormalities, metabolic disorders, autoimmune factors, and infections (Jin et al., 2012). However, approximately 50% of cases have unclear etiology, emphasizing the need for further research in this area.

Revealing the gene expression profiles and interaction networks of different cells, tissues, or organs under various conditions, and further studying the impact of genomic variation on gene expression regulation have emerged as key areas of interest (Przybyla and Gilbert, 2022). The transcription process of genes serves as the initial stage of gene expression and a critical component of gene expression regulation (Jin et al., 2012). This aspect of research plays a significant role in understanding the etiology and pathogenesis of diseases. The exploration of differential gene expression between individuals with POI and those with normal ovarian function opens a window into the molecular underpinnings of this complex condition (Yang et al., 2021).

In this study, the focus was directed toward identifying genes that exhibit altered expression patterns in women with POI compared to their normal counterparts. The results revealed a compelling disparity in gene expression levels, with 10 genes displaying upregulation and 29 genes exhibiting downregulation in the POI group. This significant observation not only highlights potential biomarkers for the condition but also hints at the intricate molecular changes that may contribute to POI pathogenesis.

Among the 39 differentially expressed genes in POI, their functions appear to be closely related to promoting cell proliferation and inhibiting cell apoptosis, suggesting their potential involvement in various stages of ovarian function. Gene set enrichment analysis unveiled significant enrichment in cellular components and molecular function. In term of cellular components, the differentially expressed genes were notably enriched in the haptoglobin-hemoglobin complex and hemoglobin complex. The molecular function analysis revealed significant enrichment in haptoglobin binding and oxygen carrier activity. Haptoglobin (Hp) is a glycoprotein found in blood plasma that binds to free hemoglobin (Hb). It plays a critical role in tissue protection and the prevention of oxidative damage (di Masi et al., 2020). Elevated serum levels of haptoglobin are often observed in response to stress conditions, such as myocardial infarction, tumor, inflammation, trauma and infection, and after the application of certain hormones, including corticosteroids and androgens (Naryzhny et al., 2021). The serum level is known to be associated with the severity and prognosis of these conditions (Naryzhny and Legina, 2021). The human ovary is one of the most susceptible organs to stress damage, with the consequent occurrence of ovarian dysfunction manifested by POI or other ovarian diseases (Petríková and Lazúrová, 2012). In POI patients, causes are related on the presence of either lymphocytic oophoritis, or other inflammation disorders (Nelson, 2009b; Gleicher et al., 2015). Furthermore, through KEGG pathway enrichment analysis, we observed that the differentially expressed genes were enriched in the biological pathway “proteoglycans in cancer”. Proteoglycans (PGs) are a group of glycoconjugates consisting of a core protein and covalently linked aminoglycans (Iozzo and Schaefer, 2015). They play essential roles in cell membranes, basement membranes, and particularly the extracellular matrix, and are closely associated with the tissue cell structure and function (Iozzo and Sanderson, 2011; Schaefer et al., 2017). Previous studies have demonstrated that proteoglycans, as amino sugars found in all tissues, exhibit complex structures and functions, including cell proliferation, migration and adhesion. PGs are important components of the matrix during follicular development, and gonadotropins increase the expression of PG-M during ovulation (Watson et al., 2012). It is hypothesized that the proteoglycan signaling pathway may be involved in altered biological behavior of endometrial cells, follicle development, and ovarian granulosa cell proliferation in POI.

In our study, we employed the support vector machine algorithm with 5-fold cross-validation to construct a classification model. By integrating clinical information with the expression levels of the top five differentially expressed genes (SLC25A39, CNIH3, PDZK1IP1, SHISA4, and LOC389834), we investigated their expression patterns and clinical significance in POI. SLC25A39 is known to play a role in mitochondrial transport and metabolism (Ruprecht and Kunji, 2020). It was reported that mitochondrial dysfunction were associated with some rare cases of POI, and manipulation of mitochondrial function represents an important therapeutic target for the treatment or prevention of POI (Tiosano et al., 2019). Therefore, the gene product of SLC25A39 plays a pivotal role in mitochondrial function by facilitating the transport of glutathione into mitochondria, which is essential for antioxidant defense and maintaining cellular redox balance (Slabbaert et al., 2016). Dysfunction in this transport mechanism, regulated by AFG3L2 and iron, may compromise mitochondrial health (Shi et al., 2024), resulting in oxidative stress within oocytes and potentially contributing to the development of POI. This underscores the significance of SLC25A39 in preserving oocyte quality and sustaining cellular energy metabolism critical for fertility. CNIH3, a gene associated with protein binding and modulation of ion channel activities (Shanks et al., 2014), shows differential expression in POI patients. While the primary function of the CNIH3 gene involves regulating AMPA receptor activity in the brain, its potential indirect influence on POI warrants consideration. Disruptions in CNIH3 signaling pathways may impact the hypothalamic-pituitary-gonadal axis, potentially leading to metabolic or oxidative stress (Challenor et al., 2015), both of which are implicated in the decline of oocyte quality and the onset of POI. However, direct evidence linking CNIH3 to POI remains limited, necessitating further research to elucidate the gene’s potential role in ovarian function and the development of POI. PDZK1IP1, also known as PAR3, is intriguing due to its role in cell polarity and cytoskeletal organization (Buckley and St Johnston, 2022). The PDZK1IP1 gene encodes a protein involved in cellular signaling pathways, particularly in organizing the cytoskeleton and mediating cell-cell interactions (Ikeno et al., 2019). Although the direct association between PDZK1IP1 and POI requires further investigation, its role in cellular processes suggests a potential involvement in maintaining ovarian function. Considering the critical role of the cytoskeleton in oocyte development and follicular maturation, any disruption due to PDZK1IP1 dysfunction could impair these processes, contributing to POI development. Additionally, PDZK1IP1’s involvement in signaling cascades related to stress response suggests that variations in this gene could impact ovarian reserve and the response to gonadotropins (Carnero, 2012), potentially influencing POI etiology. Further research is needed to elucidate the precise role of PDZK1IP1 in POI pathogenesis. SHISA4, a gene associated with cell adhesion and migration (Pei and Grishin, 2012), exhibits differential expression in the context of POI. The SHISA4 gene participates in cell differentiation and developmental processes, with its protein products regulating cell signaling pathways. This involvement in cell development hints at a potential influence on ovarian function. Since normal oocyte development and follicular maturation are vital for maintaining ovarian reserve and fertility, SHISA4 alleles affecting these processes could be involved in the development of POI. Furthermore, if SHISA4 influences signaling pathways governing oocyte growth or response to hormonal cues, it may indirectly contribute to POI. Further investigation is warranted to elucidate the precise role of SHISA4 in POI pathogenesis. LOC389834, a long non-coding RNA, emerges as a potential player in POI due to its differential expression. Long non-coding RNAs have gained attention for their regulatory roles in gene expression (Xiao and Xu, 2009). Variants within gene loci, such as LOC389834, may harbor regulatory elements or unannotated genes that influence complex traits like POI. It is conceivable that variants within LOC389834 could indirectly impact the risk of developing POI. If a gene variant within LOC389834 modulates the expression or function of genes pivotal to ovarian health—such as those involved in follicular development, hormone regulation, or cellular stress responses—it could potentially contribute to the etiology of POI. Further investigation into the functional significance of LOC389834 variants is necessary to ascertain their role in POI pathogenesis.

In the pursuit of enhancing the classification of POI, our study harnessed the potential of both gene expression profiles and hormone levels. The effectiveness of this model was quantified through the generation of a receiver operating characteristic curve, which revealed an encouraging area under the curve value of 0.83. This initial step showcased the potential of gene expression profiles to aid in the accurate classification of POI. To further refine this classification approach, we identified a set of top five differentially expressed genes—SLC25A39, CNIH3, PDZK1IP1, SHISA4, and LOC389834. Incorporating these genes into the classification model led to a notable improvement, as the AUC value rose to 0.88. These findings underscore the significance of these specific genes in characterizing the genetic basis of POI and their potential to serve as valuable biomarkers for more precise disease classification. This work not only advances our understanding of the complex interplay between genetics and hormones in POI but also holds promise for improved diagnostic precision and individualized management strategies for individuals affected by this condition.

In synopsis, this study aimed to delve into the transcriptomic profile of POI, shedding light on the gene expression alterations underpinning this condition. The research underscores the potential significance of the identified genes in elucidating the genetic basis of POI. Importantly, these genes hold promise as valuable biomarkers, potentially enhancing the accuracy of disease classification. By thoroughly exploring the transcriptomic landscape of POI, this study brings us closer to unraveling the intricate molecular mechanisms behind this condition and opens avenues for more precise diagnostic and therapeutic approaches.

## Limitation

Despite the potential for future research to clarify the functional roles of these genes and their possible use as therapeutic targets, there are significant limitations to the current study that must be recognized. There is a pressing need for more robust efforts to thoroughly understand the genetic underpinnings of POI. Additionally, upcoming studies should employ comprehensive multi-omics strategies and wider-ranging analyses to identify new biomarkers, pathways, and regulatory networks implicated in POI. This would shed light on the complex interactions between genetic, epigenetic, and environmental determinants in the development of the disease. To substantiate the results concerning the differential expression of genes observed in this research, it is essential to conduct experimental validations and to undertake larger-scale investigations with increased sample sizes.

## Conclusion

In summary, the identified genes could play a crucial role in characterizing the genetic foundation of POI, potentially serving as valuable biomarkers for enhancing disease classification accuracy.

## Data Availability

The datasets presented in this study can be found in online repositories. The names of the repository/repositories and accession number(s) can be found below: https://db.cngb.org/cnsa/, CNP0004882.
